# Factors associated with age of mother at first birth in Albania: application of quantile regression model

**DOI:** 10.1016/j.heliyon.2021.e06547

**Published:** 2021-03-23

**Authors:** Ashis Talukder, Zahidul Islam Khan, Fatheha Khatun, Shafia Tahmida

**Affiliations:** Statistics Discipline, Khulna University, Khulna, 9208, Bangladesh

**Keywords:** Age of mother at first birth, Factors, Quantile regression, Albania

## Abstract

The objective of this study was to explore the risk factors that can determine the age of mother at first birth in Albania. The necessary information was extracted from a nationally representative sample survey, Albania Demographic and Health Survey, 2017-18 dataset. To identify the possible risk factors of the age of mother at first birth, we applied quantile regression model. The mean age of mother at first birth was found to be 22.38 years with standard deviation of 3.56 years. The minimum and maximum age was reported 15 and 45 years, respectively. From the result of quantile regression, respondent's current age, education level and partner's higher education level were found positive impact on age of the mother at first birth. However, rural area, partner's age and smoking status found negative impact on age of the mother at first birth. This study suggests that women's education should be more prioritized because it can interfere with the idea of early marriage. Awareness can also be raised by social activities since the improvement of social conditions and reduction of social deprivation can increase the age of mother at first birth.

## 1 Introduction

Childbirth is said to be the most intriguing experience of a woman's life. It is an experience with physical, psychological, social and existential impact. The impact could be in both long term and short term and can be positive and empowering or negative and traumatizing (Aune et al., 2015). These days women can access a lot of information about childbirth, but still are not well prepared. They attempt to prepare for the unexpected with just information. The information they get about birth doesn't really make them understand birth or create any meaningful knowledge but increases stress (Kay, 2016).

The age of mother at first birth refers to the age when a woman conceives and gives birth to a child for the first time. The first birth is a significant incident that takes a woman into motherhood and influences her reproductive life span as fertility is directly related with it (Rabbi and Kabir, 2013). It has a huge impact on the number of children she is going to have throughout her life (Mathews and Hamilton, 2009). Even if there were positive changes in this regard in the past few years, the age at first birth is being marked as an indicator of greater social problem and even considered as a human rights issue (UNFPA, 2013a).

Almost 17 million teenage girls give birth each year which is 11% of all births worldwide (WHO, 2014). Studies have found out that, the median age at marriage in South Asia is very low, which has a huge impact on the age of mother at first birth, the examples include Bangladesh (14.1 years) and India (16.1 years) (WHO, 2014). The age of mother at first birth can affect the health of the woman in the long run and the health of her children (Maitra and Pal, 2004). In a study, it has been seen that completed fertility is reduced by 3% for females and 3.4% for males because of an additional delay of one year in childbearing (Kohler et al., 2001). The young mother may drop out of school and may not gain vocational skills which reduce the economic status that affects both herself and her child as well as the family (UNFPA, 2013b). In Eastern Europe and Central Asia, pregnancy at an early age can be put in perspective with the HIV infected people as one third of the new infections are among 15–24 age group and more than 80% of the infected are under 30 years old, 40% of which are women (UNICEF, 2010). The mortality rate is higher for those who become parents at a young age (Grundy and Kravdal, 2008). It has been seen that all-cause mortality is associated with the maternal age at first birth in a U-shaped relation (Sakai et al., 2017).

Albania is a developing country with a population of 2.8 million with the replacement rate of the total fertility is 1.5 (World Factbook, 2016). The average age of first marriage for the women was 23.6 years and 29.3 years for men in 2011 (UNECE, 2016). The society being strongly patriarchal and traditional in the rural areas of Albania, there are still arranged marriages (Young, 2002). The women are very often forced into marriages in rural areas because of patriarchal mentality and poverty, even though it is generally disapproved (IRB, 2016). The total fertility rate in Albania is different between rural and urban residence, which are 1.9 and 1.7 respectively and it is seen that total fertility rate (TFR) tends to be distributed geographically. But the women with higher education have a lower TFR in Albania. The median age of the women with no education or primary education is 21.5 years where in case of the women with higher education is 26.4 years. Wealth index also has an impact on childbearing. In Albania, 6% of teenagers with lower wealth index had initiated childbearing where only 1% were from higher quantile (Institute of Statistics, Institute of Public Health, and ICF, 2018).

**Figure 1:**
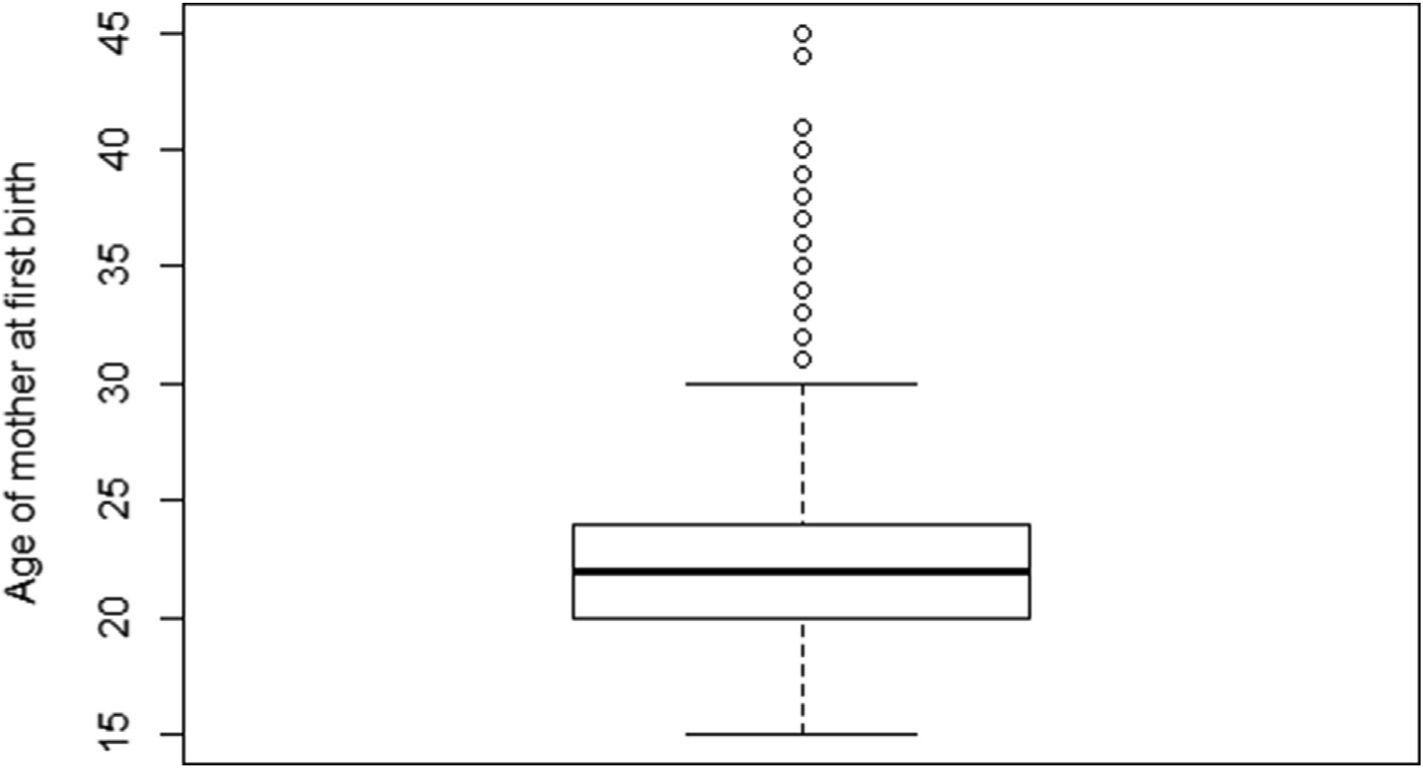
Detecting outliers of Age of mother at first birth using Box plot.

In the majority of the past examinations, the researchers attempted to identify the significant factors of fertility by investigating the age at first birth through the multiple regression or factor analysis (Hossain and Majumder, 2019). However, the quantile regression has an obvious advantage in a sense that it is robust in the presence of outliers as well as doesn't only concern itself with the mean and median behavior (Yeh et al., 2009). Moreover, the quantile regression has produced more unbiased estimates than the estimates produced by simple linear regression when the data did not follow the normality assumption (Olsen et al., 2012). The boxplot of the age at first birth displayed in Figure 1 identifies that the distribution does not follow the normality assumption. Therefore, the objective of this study is to explore the risk factors that can determine the age of mother at first birth in Albania with the application of quantile regression model.

## 2 Materials and methods

### 2.1 Data source

The data is obtained from Albania Demographic and Health Survey (ADHS), 2017–18. It is a nationally representative survey. The survey was conducted by the Institute of Public Health (IPH) and the Institute of Statistics (INSTAT) and funded by the Swiss Agency for Development and Cooperation (SDC), the United Nations Population Fund (UNFPA), The United Nations Children's Fund (UNICEF) and United Nations Entity for Gender Equality and the Empowerment of Women (UN Women). The available link of the data is https://dhsprogram.com/data/available-datasets.cfm. In the survey, as sample clusters a total of 715 enumeration areas (EAs) were selected with probability proportional to population size. In every sampling cluster 24 households were randomly selected. Some of the EAs contained fewer than 24 households, in which case all of the households were selected in those EAs. In the survey, 15,823 households were successfully interviewed. Among the households, 10,860 women aged 15–49 and 4,140 women aged 50–59 were successfully interviewed. The sample size used for the analysis was 15000, among which 6,939 women were from the urban areas and 9061 women were from the rural areas of Albania. The details of the sampling procedure are available in the report of the Albania Demographic and Health Survey, 2018 (Institute of Statistics, Institute of Public Health, and ICF, 2018).

### 2.2 Variables

In this paper, the respondent's age at first birth was considered as the main response variable. It has direct influence on a woman's cumulative fertility. The number of children a woman will have throughout her lifetime is related to the age that she has the first one. Childbearing at an earlier age can also have negative consequences on both the mother and child's health. Note that, in our study, we considered those women whose age at first birth was greater than or equal to 15 years.

Besides the main variable, a set of covariates were considered as the possible risk factors that can determine the age of mother at first birth in Albania. To explain the data in the simplest way and to avoid redundant predictors, the variables were chosen based on the previous studies and prior research experience. These variables represent demographic, socioeconomic, cultural sectors of Albania. Respondent's current age (continuous), partner's age (continuous) and body mass index (less than 18.5 = thin, between 18.5 and 24.9 = normal, greater than 24.9 = overweight) are demographic variables. The place of residence (urban, rural) and religion (Islam, orthodox, catholic, others) are cultural variables. Among the socioeconomic variables, wealth index (poor, middle, rich), partner's education (no education, primary, secondary, higher), respondent's education (no education, primary, secondary, higher) and smoking status (yes, no) were chosen for this study.

### 2.3 Statistical analysis

#### 2.3.1 Box-plot

In the case of this analysis the use of boxplot to detect normality seemed feasible because it presents information based on a five-number summary. It summarizes the data measured on an interval scale. It can be especially used for identifying whether a distribution is skewed and if there are potential outliers in the data set. In case of a large number of observations boxplot is very useful (Hossain and Majumder, 2019). It is also very easy to visualize since it can produce a graphical representation. Then based on result of the boxplot we used the quantile regression to analyze the factors behind the age of mother at first birth. The analysis was conducted using RStudio v1.2 and further rechecked by IBM SPSS Statistics Version 20.

#### 2.3.2 Quantile regression

Much of applied econometrics may be viewed as an elaboration of the linear regression model and associated estimation methods of ordinary least squares (OLS) and least absolute deviation (LAD). It is well known that the former method estimates this by minimizing the sum of the squared errors and results in an approximation to the mean function of the conditional distribution of the regressand. The later method minimizes the sum of absolute errors and fits medians to a linear function of covariates. A useful feature of the quantile regression is distinct from them as not binding that represents a central tendency of a distribution. We could go further and compute several different regression curves corresponding to the various percentage points of the distributions and thus get a more complete picture of the set. As far as the entire conditional distribution is concerned, it is not satisfactory to characterize only the mean (or median) behavior. In other words, quantile regression is robust to the presence of outliers.

**Figure 2:**
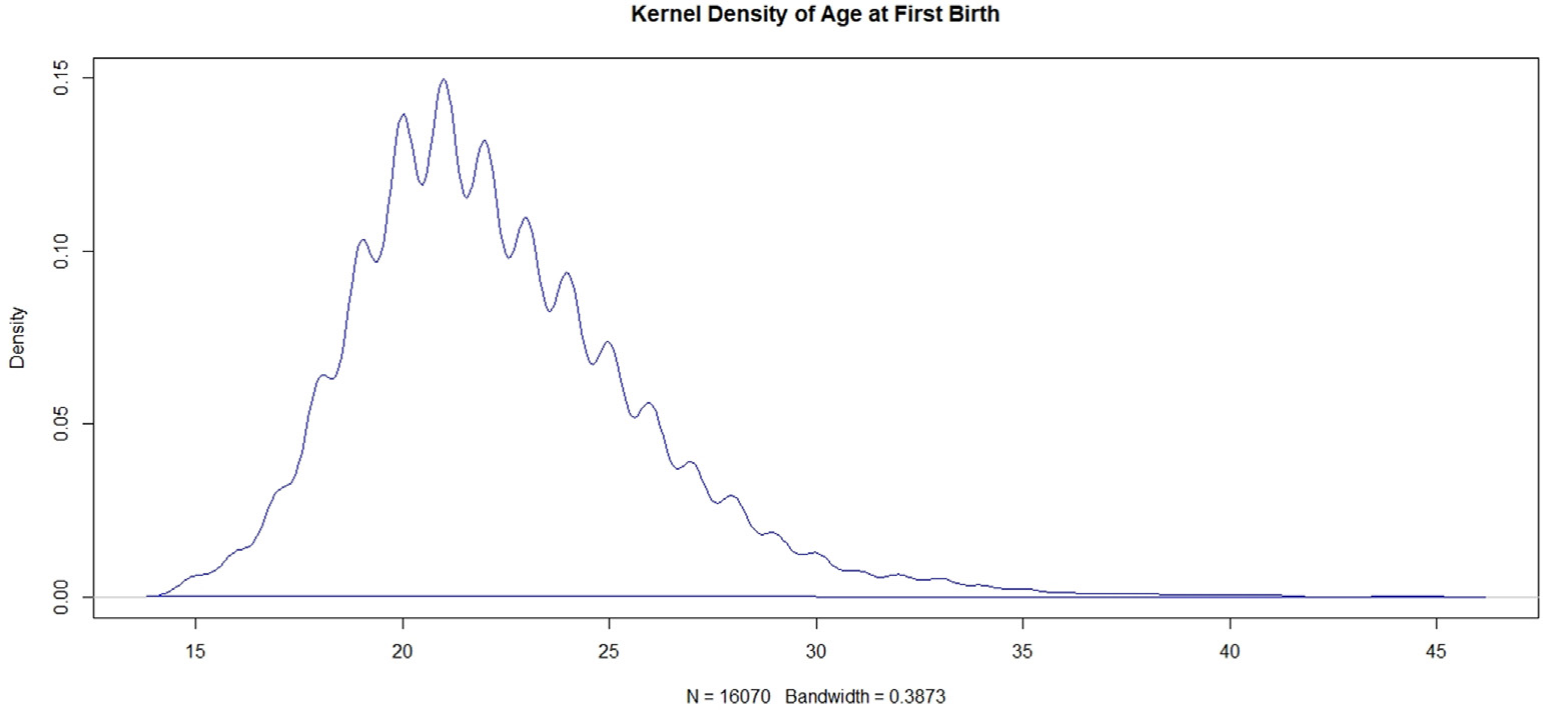
Distribution of age of mother at first birth.

The response variable of this study, respondent's age at first birth was found to be skewed and has outliers. The density plot of the variable shows positive skewness and confirms the presence of outliers. Therefore, the quantile regression method is appropriate for this study and it can provide a more precise result (see Figure 2).

The quantile regression was first proposed by Koenker and Bassett (1978). The covariate effects can be understood in a better way by quantile regression as it can estimate a family of conditional quantile functions. Given that any real-valued random variable X may be characterized by its distribution function as

(i) F(x)=Pr(X≤x)

the $\theta^{th}$ quantile is defined as

(ii) Q(θ)=inf{x:F(X)≥θ}

for 0<θ<1, X being a random variable with the distribution function given by Eq. (i).

The definition of quantile regression says that an observation in the $\theta^{th}$ percentile is greater than θ% of the observations and smaller than (1−θ)% of the observations.

Let, $\left(y_{i},x_{i}\right),i=1,2,\ldots ,n$, be a sample from some population, where $y_{i}$ is a real outcome variable of interest and $x_{i}$ is a vector of regressor including policy variables. The linear form of the general quantile regression described in Buchinsky (1998),

(iii) $$y_{i}=x_{i}\beta_{\theta }+\varepsilon_{\theta }$$

for (i=1,2,…,n), where β is a (k×1) vector of coefficients, $x_{i}$ is the column vector that is the transposition of the $i^{th}$ row of the $X_{n\times k}$ matrix of explanatory variables, $y_{i}$ is the $i^{th}$ observation of the dependent variable and $\varepsilon_{\theta }$ is the unknown error term. The $\theta^{th}$ conditional quantile of y given x can be written as

(iv) $$Quant_{\theta }=\left(y_{i}|x_{i}\right)=x_{i}\beta_{\theta }$$

Its estimate is given by $x_{i}^{\prime }\beta_{\theta }$. As θ increases continuously, the conditional distribution of y given x is traced out. Then, it is assumed that the conditional quantile of $y_{i}$, conditional on $x_{i}$, satisfies $Quant_{\theta }=\left(y_{i}|x_{i}\right)=x_{i}^{\prime }\beta_{\theta }$, for several different values of θ, θε(0,1), so that $Quant_{\theta }=\left(y_{i}|x_{i}\right)=0$. In this way quantile regression allows for parameter heterogeneity across different types of regressor. The estimator of quantile regression can be found in the following minimization problem,

(v) $$min_{\beta \varepsilon R^{k}}\underset{i\in \left\{i:y_{i}\right\}\geq x_{i}\beta }{\sum }\theta \left|y_{i}-x_{i}^{\prime }\beta_{\theta }\right|+\underset{i\in \left\{i:y_{i}\right\}\geq x_{i}\beta }{\sum }\left(1-\theta \right)\left|y_{i}-x_{i}^{\prime }\beta_{\theta }\right|$$

#### 2.3.3 Ethical statement

This study utilized ADHS dataset that is publicly available. The Institute of Public Health (IPH) and the Institute of Statistics (INSTAT) were jointly given the approval of ADHS dataset. ICF Macro Institutional Review Board in Calverton, Maryland, USA also provided partial technical assistance to approve the project. Therefore, this study does not need any additional ethical approval since the analysis was based on the freely available secondary data.

## 3 Results and discussion

### 3.1 Results

The mean age of mother at first birth was found to be 22.38 years with standard deviation of 3.56 years. It is observed that the minimum and maximum age was 15 and 45 years, respectively. The skewness of the response variable (1.03) confirmed that the distribution was positively skewed, indicating the violation of normality assumption. Moreover, the boxplot (see, Figure 1) also provides evidence that the distribution of mother's age at first birth did not follow the normal distribution. This evidence implies that the quantile regression approach would be suitable for this dataset.

Among the respondents, 0.8% had no education and 59.6%, 25.1% and 14.5% had primary, secondary and higher education, respectively. Considering partner's education, 1.8% received no education, 53.1% received primary education, 36.9% received secondary education and 8.2% received higher education. More than half of the participants (58.5%) were residing in the rural area. Most of the participants (62.8%) were in the overweight category of body mass index (BMI) measurement scale. Among wealth index, more than half of the participants fall in the poor (56.2%) category. Considering religion, 81.7% participants were believed in Islam. Only 3.2% participants were found to be smoker. In addition, respondent's mean age was found to be 38.53 years with a standard deviation of 7.42 years and her partner's mean age was observed to be 44.14 years with a standard deviation of 8.06 years. It should be mentioned that the percentages are weighted according to the proper survey weight.

In the regression analysis, we have applied quantile regression model for different quantiles ($Q_{10},Q_{20},Q_{30},Q_{40},Q_{50},Q_{60},Q_{70},Q_{80},and\;Q_{90}$) and compared with multiple regression model by using AIC (Akaike Information Criterion) value. It is an estimator of out-of-sample prediction error and along these lines' relative nature of measurable models for a given arrangement of data. Given an assortment of models for the information, AIC gauges the nature of each model, comparative with every one of different models. Since the dataset contains outliers, AIC is more appropriate to use. Moreover, AIC requires less information to predict with the same level of precision. It can include all the parameters with no interaction. In this manner, AIC gives a way to display choice. Although the AIC will choose the best model from a set, it will not say anything about absolute quality. In other words, if all the models are poor, it will choose the best of a bad bunch. Therefore, once the best model is selected, running a hypothesis test should be considered to figure out the relationship between the variables in the model and the outcome of interest. The model that has minimum AIC among all the other models is considered a good model. The results of AIC were displayed in Table 1. From the table, it was observed that the quantile regression ($Q_{30}$) had lowest AIC (77825.71) value. Therefore, we have considered quantile regression model with 30^th^ quantile as the best model for determining the factors associated with mother's age at first birth in Albania. Additionally, the R-squared of the OLS estimation was found out to be 0.81 and the Pseudo R-squared of the quantile regression rounded to the nearest hundredth place were 0.83, 0.84, 0.89, 0.85, 0.85, 0.85, 0.86, 0.84, 0.87 respectively.

**Table 1:** Parameter estimates of multiple regression and quantile regression model for different quantiles with AIC values.

	OLS (P-value)	$Q_{10}$(P-value)	$Q_{20}$(P-value)	$Q_{30}$(P-value)	$Q_{40}$(P-value)	$Q_{50}$(P-value)	$Q_{60}$(P-value)	$Q_{70}$(P-value)	$Q_{80}$(P-value)	$Q_{90}$(P-value)
Intercept	17.31 (0.00)	12.708 (0.00)	13.971 (0.00)	16.750 (0.00)	17.941 (0.00)	19.000 (0.00)	18.896 (0.00)	19.954 (0.00)	20.379 (0.00)	20.340 (0.00)
Respondent's current age	0.30 (0.00)	0.270 (0.00)	0.294 (0.00)	0.289 (0.00)	0.294 (0.00)	0.312 (0.00)	0.319 (0.00)	0.329 (0.00)	0.321 (0.00)	0.340 (0.00)
Type of place of residence										
Urban (ref)										
Rural	-0.41 (0.00)	0.100 (0.04)	-0.007 (0.90)	-0.198 (0.00)	-0.294 (0.00)	-0.437 (0.00)	-0.562 (0.00)	-0.780 (0.00)	-0.991 (0.00)	-1.237 (0.00)
Religion										
Islam (ref)										
Orthodox	0.29 (0.02)	0.215 (0.02)	0.453 (0.01)	0.471 (0.00)	0.235 (0.01)	0.187 (0.31)	0.182 (0.29)	0.040 (0.74)	0.078 (0.72)	0.381 (0.12)
Catholic	0.65 (0.00)	0.410 (0.00)	0.482 (0.00)	0.460 (0.00)	0.529 (0.00)	0.500e (0.00)	0.680 (0.00)	0.619 (0.00)	0.779 (0.00)	1.113 (0.00)
Others	-0.19 (0.26)	-0.37 (0.09)	-0.438 (0.06)	-0.045 (0.87)	-0.058 (0.68)	-0.0625 (0.72)	-0.191 (0.05)	-0.408 (0.00)	-0.402 (0.003)	-0.134 (0.75)
Wealth index combined										
Poor (ref)										
Middle	-0.26 (0.00)	0.055 (0.20)	-0.093 (0.18)	-0.051 (0.44)	-0.176 (0.005)	-0.312 (0.00)	-0.325 (0.00)	-0.458 (0.00)	-0.595 (0.00)	-0.587 (0.00)
Rich	-0.50 (0.00)	-0.157 (0.02)	-0.107 (0.14)	-0.113 (0.10)	-0.235 (0.00)	-0.437 (0.00)	-0.458 (0.00)	-0.627 (0.00)	-0.929 (0.00)	-1.154 (0.00)
BMI										
Thin (ref)										
Normal	0.64 (0.02)	0.872 (0.004)	0.733 (0.00)	0.255 (0.41)	0.411 (0.09)	0.375 (0.04)	0.552 (0.001)	0.621 (0.21)	0.644 (0.29)	0.896 (0.00)
Overweight	0.16 (0.54)	0.447 (0.14)	0.388 (0.05)	-0.073 (0.81)	0.058 (0.81)	0.00 (0.00)	0.515 (0.38)	0.137 (0.78)	-0.013 (0.98)	0.278 (0.10)
Partner's age	-0.21 (0.00)	-0.200 (0.00)	-0.230 (0.00)	-0.232 (0.00)	-0.235 (0.00)	-2.50e-01 (0.00)	-0.249 (0.00)	-0.254 (0.00)	-0.214 (0.00)	-0.195 (0.00)
Partner's education level										
No education (ref)										
Primary	-0.57 (0.01)	0.472 (0.15)	-0.122 (0.81)	-0.090 (0.57)	-0.470 (0.17)	-0.500 (0.06)	-0.519 (0.04)	-0.730 (0.00)	-1.248 (0.01)	-2.412 (0.01)
Secondary	-0.54 (0.01)	0.703 (0.03)	0.007 (0.99)	-0.068 (0.67)	-0.470 (0.52)	-4.37e-01 (0.11)	-0.610 (0.02)	-0.872 (0.00)	-1.304 (0.009)	-2.206 (0.02)
Higher	0.18 (0.42)	1.184 (0.00)	0.748 (0.15)	0.687 (0.00)	0.235 (0.00)	3.75e-01 (0.21)	0.325 (0.24)	-0.131 (0.88)	-0.523 (0.31)	-1.855 (0.05)
Respondent's education										
No education (ref)										
Primary	2.90 (0.00)	3.039 (0.00)	3.827 (0.00)	2.602 (0.00)	2.294 (0.00)	2.060 (0.00)	2.574 (0.00)	2.532 (0.00)	2.746 (0.00)	4.010 (0.01)
Secondary	3.63 (0.00)	3.751 (0.00)	4.446 (0.00)	3.318 (0.00)	3.117 (0.00)	2.810 (0.00)	3.325 (0.00)	3.456 (0.00)	3.545 (0.00)	4.443 (0.008)
Higher	4.91 (0.00)	4.492 (0.00)	5.323 (0.00)	4.289 (0.00)	4.294 (0.00)	4.180 (0.00)	4.759 (0.00)	4.958 (0.00)	5.068 (0.00)	6.650 (0.00)
Smoking status										
No (ref)										
Yes	-1.07 (0.00)	-1.526 (0.00)	-1.410 (0.00)	-1.227 (0.00)	-0.941 (0.00)	-1.00 (0.00)	-1.006 (0.00)	-0.842 (0.01)	-0.942 (0.00)	-0.463 (0.40)
AIC	80006.5	80411.03	78355.45	77825.71	78132.16	79099.13	80656.66	83048.61	86624.36	92608.87
Pseudo R-squared	0.81	0.83	0.84	0.89	0.85	0.85	0.85	0.86	0.86	0.87

From the result of quantile regression ($Q_{30}$), we identified that respondent's current age (0.289) is positively associated with the age of mother at first birth which has a significant p-value (p<0.001). The respondents residing in rural area have a negative (Estimate = -0.198) impact on age of the mother at first birth which means that the women residing in rural area, the first birth age of mother is significantly (p<0.001) lower than the women residing in urban area. Similarly, for religion, Orthodox (Estimate = 0.471; p<0.001) and Catholic (Estimate = 0.460; p<0.001) has positive impact on age of the mother at first birth. In addition, partner's age (Estimate = -0.232;p<0.001) is negatively associated with the age of mother at first birth. Partner's higher education level (Estimate = 0.687;p<0.001) has positive impact on age of the mother of first birth. Similarly, the three categories of the respondent's highest education level primary (Estimate = 2.602;p<0.001), secondary (Estimate = 3.318;p<0.001) and higher (Estimate = 4.289; p<0.001) had positive impact on age of mother of first birth. The smoking status (Estimate = -1.227;p<0.001) has a negative impact on the age of the mother at first birth.

### 3.2 Discussion

The aim of our study was to find out the underlying factors associated with the age of mother at first birth among Albanian women using the ADHS, 2017-18 dataset. The quantile regression model was used because it can be used even if the data contain outliers and provides more unbiased and accurate result compared to the linear regression model. The quantile regression model was compared to the multiple linear regression model using AIC and the significance of the variables were determined by the p-values. Among the models, using AIC, the 30^th^ quantile was found to provide the best possible estimates. The results of the study suggest that the place of residence, religion, partner's age, partner's education level, respondent's highest education level, smoking status are the significant risk factors of the age at first birth of Albanian women.

In our study we found that teenage girls who live in rural areas are more likely to step into motherhood earlier than the girls living in urban areas. This findings coincide with other previous studies (Hossain and Majumder, 2019; Islam et al., 2017; Rabbi and Kabir, 2013). The women from the rural areas of Albania tend to conceive earlier. The socioeconomic gap between the urban and rural areas could be the reason behind it. The lack of education in the rural areas may also affects it.

Religion also affects the age of mother at first birth (Hossain and Majumder, 2019). The women who follow Islam are found to get into motherhood earlier than the followers of other religions (Jiloha, 2009). We have found that the Albanian women who follow Islam also conceive earlier than the women who follow Catholic or Orthodox, which shows the impact of religion on adolescent motherhood. Moreover, our research finds that the partner's age have significant negative effect on age at mother at first birth. However, a previous study reported an opposite result conducted in Bangladesh (Hossain and Majumder, 2019). There may be a regional effect for this type of findings, although need further study.

This study identifies that both respondent's as well as partner's education level have significant positive effects on age of mother at first birth. Several previous study also reported similar result (Hossain and Majumder, 2019; Islam et al., 2017; Rabbi and Kabir, 2013). The women who lack proper education have no adequate knowledge about the high risk of being pregnant early. They also lack the knowledge of family planning and risk on their health as well as their children. Another fact that arises with the lower education level is that these women have lower empowerment within the family (Angeles et al., 2005; Islam, 2014; Marchetta and Sahn, 2016). Therefore, the increase in the level of education level can be a better solution to increase the first birth age of mothers in Albania.

Our study explore that the smoking status has a direct effect on the age of mother at first birth. A previous study also reported similar result (Hossain and Majumder, 2019). Women who developed the habit of smoking at a young age (under 16) had their first child earlier than other. However, we need further investigation to explore the exact reasons that exist behind this findings.

## 4 Conclusion

First birth is an important phase of a woman's life. It is also a risk factor of the mother's health and fertility. This study was conducted to find out the risk factors of a woman's age at first birth. From the results that were found in the study we can conclude there lies some socio-economic factors behind the problem as well as education is a key factor. Even religion plays a great role behind the age of mother at first birth. Though adolescent childbearing has decreased significantly over the years, it's still not enough. We can recommend some initiatives that can be implemented by the government, general people or NGO's and development organizations to increase the age of mother at first birth. First of all, increasing the age of marriage can be suggested. Since early pregnancies will not be unwanted in early marriages because the couple become anxious to prove their fertility and the families also expect that, thus it can be suggested to increase the age of marriage. The study suggests that women's education should be more prioritized because it can interfere with the idea of early marriage. Education can empower women and create awareness about the problems of early marriage and the risks of being mother at a young age. Additionally, sexuality education should also be introduced. It should not be considered a taboo. In many developed countries sex education is a part of primary or secondary schools and thus the girls become more self-aware. Awareness can also be raised by social activities. The unlimited power of media can be of great use in this matter. In many cases early childbearing is related to low levels of income. Social deprivation is an important causal factor in such cases. The improvement of social conditions and reduction of social deprivation can increase the age of mother at first birth.

## Declarations

### Author contribution statement

Ashis Talukder, Zahidul Islam Khan: Conceived and designed the experiments; Analyzed and interpreted the data; Contributed reagents, materials, analysis tools or data; Wrote the paper.

Fatheha Khatun, Shafia Tahmida: Performed the experiments; Analyzed and interpreted the data.

### Funding statement

This research did not receive any specific grant from funding agencies in the public, commercial, or not-for-profit sectors.

### Data availability statement

Data associated with this study has been deposited at https://dhsprogram.com/data/available-datasets.cfm.

### Declaration of interests statement

The authors declare no conflict of interest.

### Additional information

No additional information is available for this paper.
